# Spatio-Temporal Dynamics of Yeast Mitochondrial Biogenesis: Transcriptional and Post-Transcriptional mRNA Oscillatory Modules

**DOI:** 10.1371/journal.pcbi.1000409

**Published:** 2009-06-12

**Authors:** Gaëlle Lelandais, Yann Saint-Georges, Colette Geneix, Liza Al-Shikhley, Geneviève Dujardin, Claude Jacq

**Affiliations:** 1 Dynamique des Structures et Interactions des Macromolécules Biologiques (DSIMB), INSERM UMR-S 665, Université Paris Diderot, Paris, France; 2 Laboratoire de Génétique Moléculaire, CNRS UMR 8541, Ecole Normale Supérieure, Paris, France; 3 MTI, INSERM UMR-S 973, Université Paris Diderot, Paris, France; 4 Laboratoire de Génétique Moléculaire, CNRS, Gif/Yvette, France; EMBL, Germany

## Abstract

Examples of metabolic rhythms have recently emerged from studies of budding yeast. High density microarray analyses have produced a remarkably detailed picture of cycling gene expression that could be clustered according to metabolic functions. We developed a model-based approach for the decomposition of expression to analyze these data and to identify functional modules which, expressed sequentially and periodically, contribute to the complex and intricate mitochondrial architecture. This approach revealed that mitochondrial spatio-temporal modules are expressed during periodic spikes and specific cellular localizations, which cover the entire oscillatory period. For instance, assembly factors (32 genes) and translation regulators (47 genes) are expressed earlier than the components of the amino-acid synthesis pathways (31 genes). In addition, we could correlate the expression modules identified with particular post-transcriptional properties. Thus, mRNAs of modules expressed “early” are mostly translated in the vicinity of mitochondria under the control of the Puf3p mRNA-binding protein. This last spatio-temporal module concerns mostly mRNAs coding for basic elements of mitochondrial construction: assembly and regulatory factors. Prediction that unknown genes from this module code for important elements of mitochondrial biogenesis is supported by experimental evidence. More generally, these observations underscore the importance of post-transcriptional processes in mitochondrial biogenesis, highlighting close connections between nuclear transcription and cytoplasmic site-specific translation.

## Author Summary

In bacterial and eukaryotic cells, gene expression is regulated at both the transcriptional and translational levels. In eukaryotes these two processes cannot be directly coupled because the nuclear membrane separates the chromosomes from the ribosomes. Although the transcription levels in different cellular conditions have been widely examined, genome-wide post-transcriptional mechanisms are poorly documented and therefore, the connections between the two processes are difficult to explain. In this work, the time-regulated expression of the genes involved in the construction of the mitochondrion, an important organelle present in nearly all the eukaryotic cells, was scrutinized both at transcriptional and post-transcriptional levels. We observed that temporal transcriptional profiles coincide with groups of genes which are translated at specific cellular loci. The description of these relationships is functionally relevant since the genes which are transcribed early in mitochondria cycles are those which are translated to the vicinity of mitochondria. In addition, these early genes code for essential assembling factors or core elements of the protein complexes whereas the peripheral proteins are translated later in the cytoplasm. Also, these observations support the concerted action of important regulatory factors which control either the gene transcription level (transcription factors) or the mRNA localization (mRNA-binding proteins).

## Introduction

Cell construction requires the tight linking of various molecular processes, from nuclear transcription to the site-specific production of proteins. The control of the orchestration of these processes remains poorly understood. In classical experimental conditions, coordinated waves of transcription are difficult to observe because of the metabolic asynchrony of the cells in growing cultures. A yeast system with properties avoiding these difficulties was recently described. In well-defined continuous cultures of *Saccharomyces cerevisiae*, the oxygen consumption rate oscillates with a constant period [1], implying that cell-to-cell signaling synchronizes oxidative and reductive functions in the culture. The gene-expression dynamics of the yeast metabolic cycle is therefore a useful model system for studies of the lifecycle of groups of transcripts in eukaryotic cells [2]. Indeed, microarray studies have demonstrated periodicity in the expression of the yeast genome, and consequently the existence of similar temporal expression patterns in functionally connected groups of genes [3]. Genes specifying functions associated with energy appeared to be expressed with exceptionally robust periodicity, consistent with the variations in the amount of dissolved oxygen in the medium of synchronized culture. In pioneering studies [4], it was shown that yeast mitochondrial morphology oscillates in response to energetic demands driven by the ultradian clock output.

In this work, our purpose was to distinguish temporal gene clusters, which may allow describing a biologically relevant scenario of mitochondria biogenesis. Depending on the addressed points and on the quality of the microarray data, several methods such as SVD (Singular Value decomposition), PCA (Principal Components Analysis), self-organizing maps, wavelet multiresolution decomposition and FFT (Fast Fourier Transform) have been used to analyze relevant transcript data [5]. We decided to use a model-based approach [6] to decomposition of published expression data for the 626 oscillating nuclear genes encoding mitochondrial proteins. We established a classification of these genes into temporal groups, which cover the 5-hour long metabolic cycle, and present a dynamic and global picture of mitochondrial biogenesis. These temporal groups correlate both with particular functional properties of the corresponding proteins and with specific translational sites in the cell. This global description of mitochondrial transcriptome clusters in temporal phases is consistent with the concept of RNA regulons, according to which post-transcriptional RNA operons may constitute an important element of eukaryotic genome expression [7],[8].

## Methods

### Source of microarray data and gene selection

Microarray data from the study by Tu *et al*. [3] were collected from the Gene Expression Omnibus database [9], under accession number GSE3431. This dataset comprised the normalized gene expression values, *i.e.* the median of each array (all data points) equal 1, used by Tu *et al.* [3] in their pioneering analysis. Tu *et al.* [3] performed microarray experiments at 25-minute intervals, over three consecutive metabolic cycles (the length of one cycle is ∼300 minutes). For each gene, expression measurements were thus available for 36 successive time points. We considered only those genes for which expression measurements were available and which (*i*) displayed significant periodic patterns, as defined by Tu *et al.* [3] (∼3552 genes with a confidence level greater than 95%) and (*ii*) were identified as involved in mitochondrial biogenesis, as defined by Saint-Georges *et al.* [10] (∼794 genes). The resulting expression matrix comprised data for 626 genes (the complete list is available in Dataset S1).

### Model-based decomposition of periodic gene expression patterns

#### EDPM algorithm

**Figure 1:**
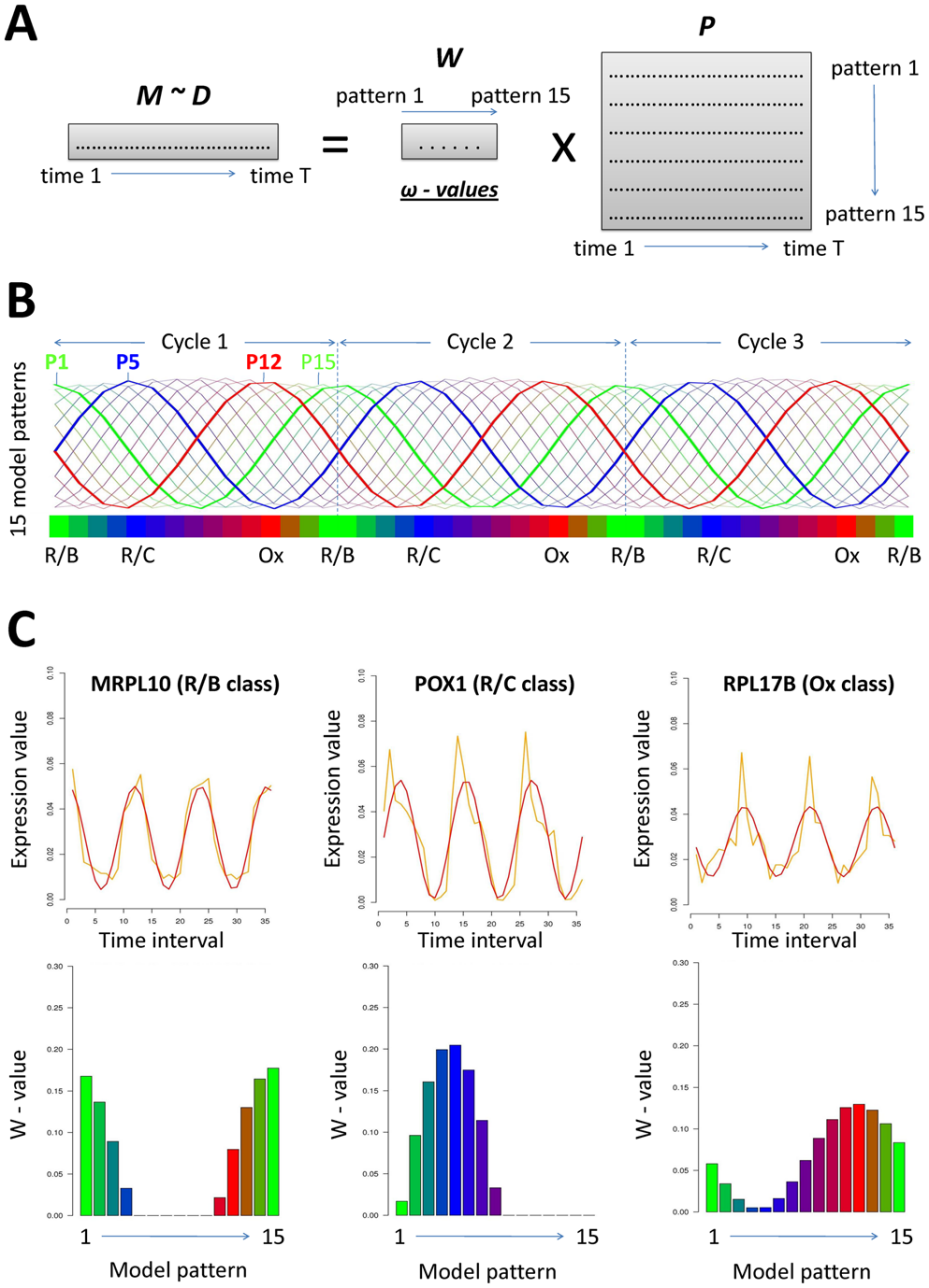
Description of the EDPM algorithm. A: Description of the vectors (***D*** *,* ***M*** and ***W***) and the matrix (***P***) used by EDPM. The algorithm calculates the vector ***W*** of ***ω-values,*** using an optimization procedure (see main text and Figure S1). B: Representation of the 15 model patterns used in this study. These models are periodic functions covering three consecutive cycles. The color code reflects the metabolic phases during which model patterns are maximal (R/B = green; R/C = blue and Ox = red). C: Illustration of EDPM results analyzing R/B, R/C and Ox sentinel genes defined in [3]. Initial vectors from the microarray data ***D*** are plotted in orange; the ***M*** vector, obtained by multiplying of ***W*** and ***P**,* is plotted in red. The **ω-*values*** (also referred to as **ω** *-*footprints in the main text) are represented as barplots and model patterns are indicated with a color code.

**Figure 2:**
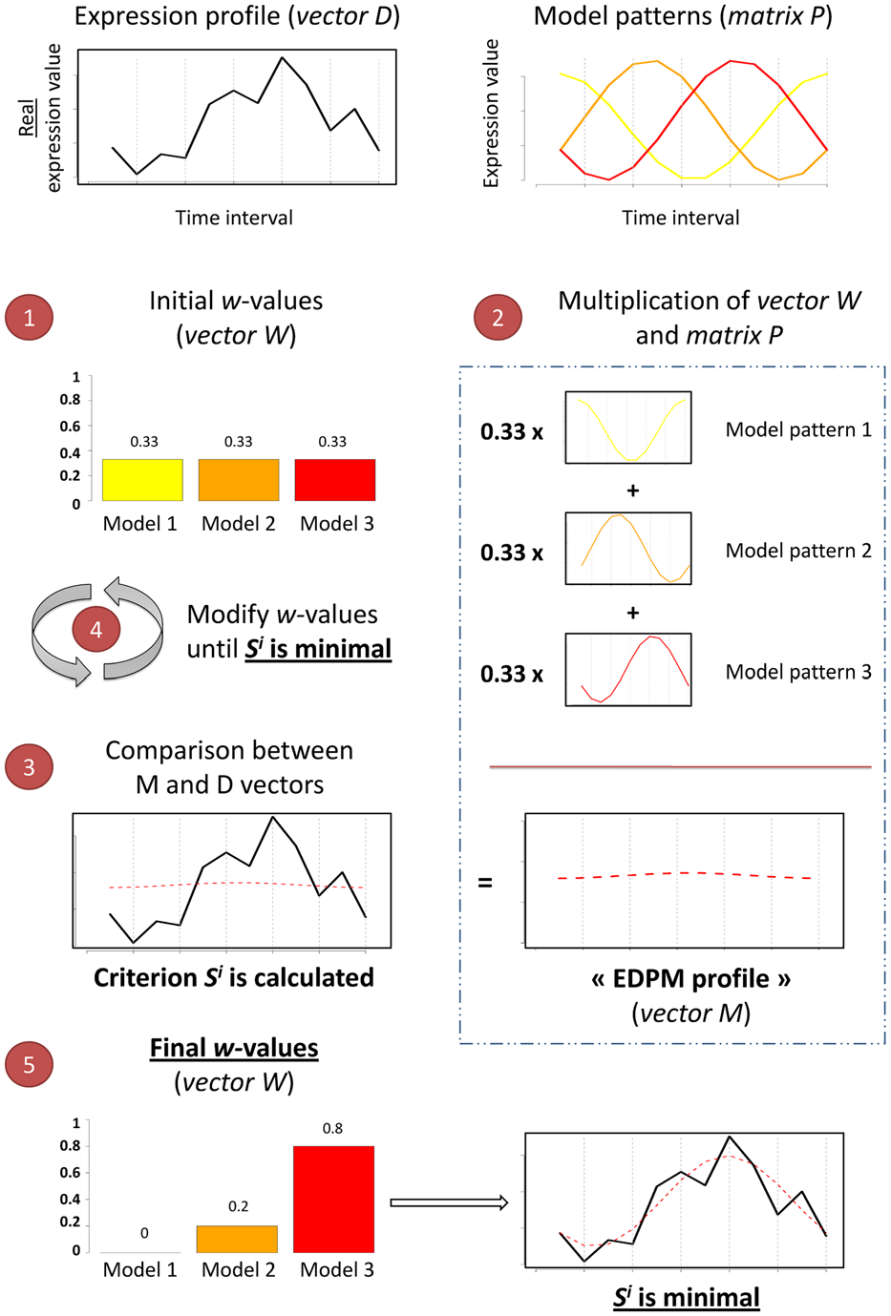
Illustration of the EDPM optimization. In this illustration, one gene expression profile is analyzed (vector ***D*** *,* black line) and 3 model patterns are used (colored respectively in yellow, orange and red). They are oscillatory functions (one cycle), with constant period but different phases. They correspond to the matrix ***P*** presented Figure 1A. The main idea is to determine the vector ***W*** of **ω*-values*** such that the square distance between the ***M*** and ***D*** vectors is minimal (*i.e.* the criterion S_i_ is minimal). (1) Each **ω*-value*** corresponds to one of the 3 model patterns and they are represented using the same color code (yellow, orange and red). The procedure is initiated with identical **ω*-values***. (2) Illustration of the standard multiplication between vector ***W*** and the model pattern matrix ***P***. The result forms a vector ***M*** called “EDPM profile” (red dashed line). (3) Vectors ***M*** (red dashed line) and ***D*** (black line) are compared, calculated the S_i_ value. (4) The **ω*-values*** are modified until the vectors ***M*** and ***D*** are as close as possible (S_i_ is minimal). To perform this optimization procedure, quasi-Newton method is used in EDPM. (5) Final results. The **ω*-values*** indicate the contribution of each model pattern to the real expression pattern. In this example, model pattern n°3 represents 80% of the observed signal, whereas model patterns n°1 and n°2 represent respectively only 0 and 20%.

Our aim was to investigate in more detail the published gene expression profiles obtained from yeast cell cultures displaying highly periodic cycles in the form of respiratory bursts. Starting from previous work [6], we developed the EDPM algorithm (Expression Decomposition based on Periodic Models) to analyze precisely gene expression patterns during yeast metabolic cycles. Overview of the EDPM procedure is presented in Figure S1. The main idea is to decompose each gene expression profile obtained with microarray technology (vector ***D***, Figure 1A), into a mixture of pre-defined model patterns (matrix ***P***, Figure 1A). For that, the algorithm calculates a vector ***W*** of **ω**-values, such that the standard multiplication of vector ***W*** and matrix ***P***, forms a vector ***M*** that reproduces the initial vector of expression measurements ***D*** (eq. 1 below). The **ω-** ***values*** are determine using an optimization procedure (see below and Figure 2 for an illustration) and therefore indicate the contribution of each model pattern to the expression pattern observed for a particular gene. The data vector ***M*** matches the vector ***D*** exactly if ***P*** is a perfect model of the biological:
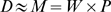
(1)


#### Optimization criterion for calculating ω-values

The ***W*** vector is calculated to minimize the square of the distance between the ***M*** and ***D*** vectors. For a given gene *i*, the criterion to be optimized — *i.e*. numerically minimized — to find the optimum solution of **ω-** ***values*** 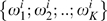 is as follows:
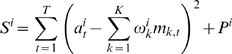
(2)Where, 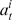 is the microarray expression measurement of gene *i* at time *t*, 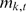 is the value of model pattern *k* at time *t*, and K is the number of model patterns (K = 15 in this study, see below). *P^i^* is a penalty function introduced to ensure that the sum of **ω-** ***values*** is equal to 1:
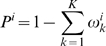
(3)Hence, the greater the value of 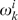 the greater the contribution of model pattern *k* to the observed expression pattern of gene *i*.

Finally, two others parameters controlling the amplitude and the level of the ***M*** vector oscillatory signals are also included in the optimization procedure (Text S1). They are not specified here for the sake of clarity.

#### Model patterns

All the genes whose expression oscillates in Tu *et al.* [3] dataset exhibit specific properties, as periodic signals among 3 successive cycles and a unique period for all genes. Using these two characteristics, we defined 15 model patterns (see Figure 1B) according to simple cosine functions, *i.e.*

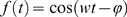
(4)Where *w* = 1 (Tu *et al.* [3] periodic patterns have a constant period) and *t* varies from 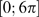 (three periods to model the three successive metabolic cycles covered by the microarray measurements of Tu *et al.* [3]). 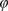 represents a time interval between the different model patterns, it varies from 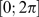 such that each model pattern reaches its maximal value a different time *t*. As all the model patterns differ only in terms of the time interval between patterns, the **ω-** ***values*** calculated by EDPM can be seen as a kind of gene “footprint”, indicating the time phase during the metabolic cycle, at which the gene is strongly expressed (and/or its mRNA is present in larger amounts).

Note that in EDPM, model patterns are pre-defined in order to match specific properties shared by the analyzed gene expression patterns. The more the model patterns are adaptable to the observed gene expression measurements, the more the EDPM optimization is efficient, *i.e.* the final 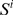 value (eq. 2) is close to 0. In the case study of the YMC biological process presented here, cosine functions appeared to be a relevant choice (Text S1). In principle, any microarray dataset can be analyzed using the EDPM approach, it may only required the definition of new model patterns adapted to the gene expression characteristics.

#### Analysis of the sentinel genes defining the successive R/B, R/C and Ox phases of the yeast metabolic cycle

As an illustration, we used the EDPM algorithm to analyze the gene expression profiles of the three sentinel genes (*MRPL10*, *POX1* and *RPL17B*) used by Tu *et al.* [3] to define three successive superclusters of gene expression during the metabolic cycle: R/B (reductive, building), R/C (reductive, charging) and Ox (oxidative). The results are shown Figure 1C. ***D*** and ***M*** vectors are superimposed and represented graphically in the upper part of panel C. The initial vector of expression measurements, ***D***, obtained with microarray technology is plotted in orange; the ***M*** vector, obtained by the multiplication of vector ***W*** (calculated with EDPM) and model pattern ***P*** is plotted in red. EDPM appears to give a smoothed representation of gene expression profiles and hence facilitates identification of the time interval in which the gene is mostly expressed. The ***ω-***derived “footprint” representations of each gene expression profile are represented in the bottom part of panel C. As expected, they are clearly different and reveal three distinct periods for maximal gene expression: successive green patterns nos. 1 and 15 for the R/B sentinel gene (*MRPL10*); blue pattern no. 5 for the R/C sentinel gene (*POX1*); and red pattern no. 12 for the Ox sentinel gene (*RPL17B*).

### Time-dependent clustering using EDPM results

To cluster genes whose RNA level peaks at the same time points in the yeast metabolic cycle (YMC), we used the **ω** ***-values*** obtained for each gene using EDPM algorithm (see previous paragraph). Pearson correlation coefficients (***r***) were calculated between all ***W*** vector pairs, and hierarchical cluster analysis was applied. This classical clustering method can be summarized as follows: (1) Distances (***d***) between all ***W*** vector pairs is calculated using Pearson's correlation analysis (***d = 1−r***); (2) The resulting distance matrix is thoroughly inspected to find the smallest distance; (3) The corresponding genes are joined together in the tree and form a new cluster; (4) The distances between the newly formed cluster and the other genes are recalculated; (5) Steps 2, 3 and 4 are repeated until all genes and clusters are linked in a final tree.

### Search for cis-acting signals in 3′ and 5′ UTR sequences

We searched for *cis*-acting signals in 3′ and 5′UTR sequences, using motifs predicted by the MatrixREDUCE algorithm [11]. For 3′UTR signals, we tested several motifs identified in previous studies [10],[12] as possible binding sites for mRNA stability regulators in *Saccharomyces cerevisiae*. For 5′ UTR signals, we examined upstream regions between nucleotide positions −600 and −1 and searched for motifs between 1 and 7 nt long. We assessed whether any of the signals were observed at a frequency greater than that expected by chance, by calculating p-values as described in [13] (hypergeometric distribution). We then search the YEASTRACT database for transcription factors with DNA-binding sites matching the motifs identified with MatrixREDUCE [14].

### Technical information

The EDPM algorithm was implemented in R programming language (http://cran.r-project.org/) and functions were numerically minimized using the quasi-Newton method (R function available in the BASE package). Hierarchical clustering was carried out with the “hclust” function (also available in R programming language), with the “ward” method for gene agglomeration. MatrixREDUCE source code is freely available online from http://bussemaker.bio.columbia.edu/software/MatrixREDUCE/ and was used for analyses of upstream sequences with default parameters (see the documentation available online for more information).

### Experimental procedures

All the strains used in this study are isogenic to BY4742 (MATα; his3Δ1; leu2 Δ0; lys2 Δ0; ura3Δ0) from the Euroscarf gene deletion library.

#### Rho^−^

To test the maintenance of the mitochondrial genome, the mutant cells were crossed with the *rho°* control strain KL14-4A/60 (*MATa his1, trp2, rho*°), devoid of any mitochondrial genome, and the diploid growth was tested on respiratory medium containing 2% glycerol.

#### Growth on non fermentable media

To test mutant respiratory growth, the cells were streaked on non-fermentable media containing 2% glycerol, 2% ethanol or 0.5% lactate and incubated for several days at 28 or 36°C.

#### Cytochrome spectra

Cytochrome absorption spectra of whole cells grown on 2% galactose were recorded at liquid nitrogen temperature after reduction by dithionite using a Cary 400 spectrophotometer (Varian, San Fernando, CA) [15].

## Results

### Periodic expression of nuclear genes involved in mitochondria biogenesis

#### Most of the genes associated with mitochondria display periodic patterns of expression during Yeast Metabolic Cycles

Tu *et al.* [3] showed that yeast cells grown under continuous and nutrient-limited conditions display highly periodic cycles (called Yeast Metabolic Cycles or YMC), in the form of respiratory bursts in which more than half the entire genome (∼3552 genes in *S. cerevisiae*) is expressed in a periodic manner. Among these numerous genes whose mRNA level is modified during YMC, we identified 626 genes as being involved in mitochondrial biogenesis. These genes account for 86% of the nuclear genes known to encode proteins found in mitochondria [10]. This observation suggests that genes associated with the mitochondria tend to display more periodic patterns of expression than the other yeast genes (p-value is lower than 1×10^−40^). This set of 626 genes was used for the following analyses.

#### Time-dependent gene clustering using EDPM algorithm

To investigate in detail the published gene expression profiles for the 626 genes involved in mitochondrial biogenesis, we developed the EDPM algorithm (Expression Decomposition based on Periodic Models, see Methods and Figure 1) for two reasons. First, we wanted to identify precisely the time interval in the YMC during which each mitochondrial gene is mostly expressed. Second, we wanted to associate mitochondrial genes into temporal classes representing distinct expression phases during YMC. The principle of the EDPM algorithm consists in breaking each gene expression pattern down into a mixture of model patterns (Figure 1A and Figure S1). These model patterns are time-delayed mathematical functions mimicking ideal expression oscillations (1 to 15, Figure 1B). EDPM allows the calculation of ω-derived “footprint” representations of each gene expression profile; these representations describe the contribution of each model pattern to the gene expression profile analyzed and hence, are good indicators of the time at which the RNA of the gene peaks in abundance during the metabolic cycle (see Figure 1C for an illustration). Note that two genes, expressed at the same moment but with different magnitudes, may exhibit identical ω-footprints with EDPM.

The 626 genes involved in mitochondria biogenesis were analyzed with EDPM (Dataset S2) and classified in several clusters according to their ω-footprints. Hierarchical cluster analysis (see Methods) objectively supported a six-cluster distribution. These clusters were named A to F. Each comprises a distinct subclass of genes that are periodically expressed and the mRNAs in each cluster peaks in different time windows of the metabolic cycle (see Dataset S3 for a complete list of genes in each cluster).

In Text S1, we present detailed justifications for the use of EDPM algorithm together with several methodological controls. Important conclusions could be raised from these analyses. In particular, we could demonstrate that a minimal number of 9 model patterns is required to stabilize the gene repartition into phases A to F. Under 9, the number of model patterns was not enough to precisely indentified the phase transitions EDPM. The choice of 15 model patterns appeared to be a good compromise between a precise determination of transition phases and the computation time required to perform EDPM decomposition. Moreover, we could observe that the EDPM criterion was significantly smaller using the real expression data rather than the sample data. This justified the use of EDPM for the 626 genes analyzed in this study. All these genes exhibit periodic gene expression profiles during the YMC (this was demonstrated by Tu *et al.* [3]) and hence are compatible with the 15 model patterns used here. Moreover, it should be noted that the final *w*-values distribution is also a good indicator of the EDPM relevance. In case of shuffle data, the *w*-values are homogeneously distributed, indicating that no particular model pattern can explain the random expression profiles (see Text S1 for an illustration). Finally, a comparative analysis of the clustering results obtained with EDPM *w*-values and other methodologies allowed us to demonstrate that EDPM improves the dissection of the expression temporal waves.

#### Clusters A to F represent different expression phases during the metabolic cycle

**Figure 3:**
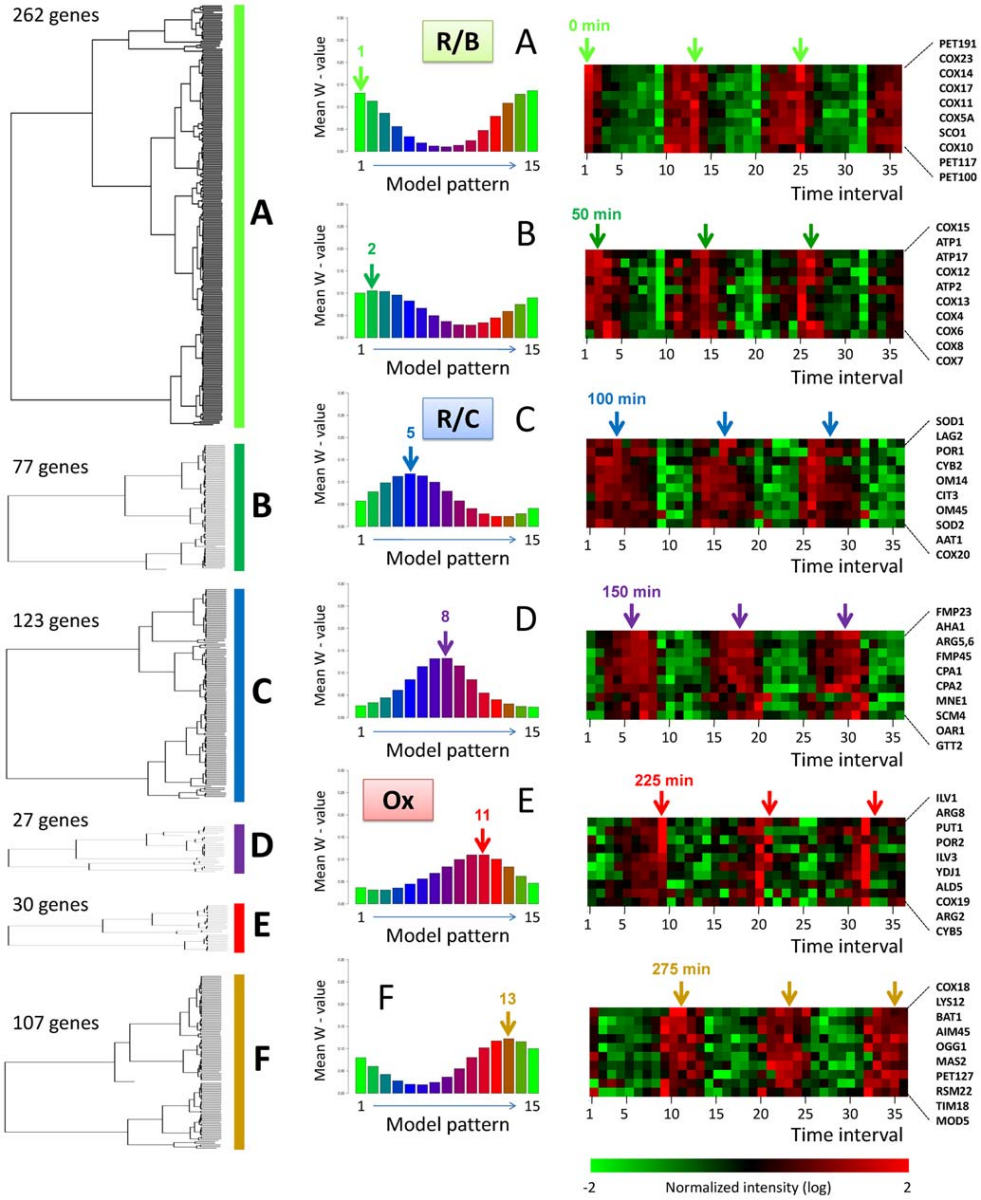
Cluster analysis of nuclear genes involved in mitochondrial biogenesis. Periodic gene expression data for yeast grown under continuous, nutrient-limited conditions [3] were analyzed using the EDPM algorithm. The ***ω***-footprints (or ***ω-values***) were calculated for each of the 626 gene expression profiles and used for hierarchical cluster analysis (left). Six clusters, A to F, account for the time course of periodic expression. They are represented along a time scale from the top (cluster A) to the bottom (cluster F). A mean *ω-*footprint is represented for each cluster (middle) and maximal values are indicated by vertical arrows together with the number of the associated model pattern. Correspondences between maximal ***ω**-* *values* and time points in the metabolic cycle are indicated by vertical arrows. Clusters A to F correspond to distinct phases during the metabolic cycle [3]: clusters A and B correspond to reductive-building (R/B); clusters C and D, to reductive-charging (R/C); and clusters E and F to oxidative activity (Ox). For each of the 6 clusters, a set of 10 typical genes is represented (left), together with their expression variations.

**Figure 4:**
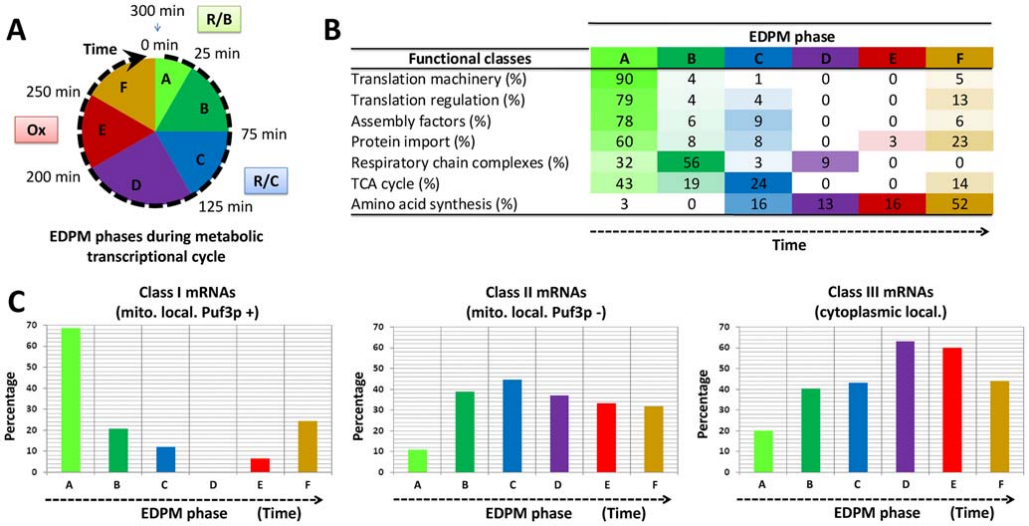
Functional and translational properties of the mRNAs in the different phases of the mitochondrial cycle. A: Correspondence between the different EDPM clusters (or phases A to F) identified in this work (Figure 3) and the major R/B, R/C and Ox phases previously identified in the 5-hour (or 300-minutes) yeast metabolic cycle [3]. The total gene content of the different phases is indicated in Figure 3 and complete list of genes is available in Dataset S3. Note that phase A lasts for only 25 min and contains 262 genes (41% of the 626 nuclear genes coding for mitochondrial proteins). B: Distribution of 7 important functional families (extracted from Dataset S4) across the temporal phases A to F. The number of genes follows the functional class name and, for each phase, the percentage (%) of genes is indicated. C: Translational properties of the 626 nuclear genes in the clusters A to F. Three translational groups of genes have recently been described [10] (see also the schematic representation in Figure 6A). Class I mRNAs are translated on mitochondria-bound polysomes, and this localization depends on the RNA binding protein Puf3p. Class II mRNAs are translated on mitochondria-bound polysomes, this localization does not depend on Puf3p. Class III mRNAs are translated on free cytoplasmic polysomes. The distribution (%) of members of the six phases A to F in the three translational classes shows that most phase A mRNAs are in class I. The color code refers to previously published work [3] describing the temporal compartmentalization of the whole genome: green = R/B, blue = R/C, red = Ox. Note that our phase analysis generally agrees with this previous work, but it is more precise and distinguishes biologically coherent groups of genes. For instance, we split the R/B phase into phases A and B, which clearly correspond to genes with different translational and transcriptional properties (see the main text).

The gene clusters presented in the previous section can be distinguished by the model patterns that contribute the most to the EDPM decompositions (see vertical arrows, middle panel Figure 3). The 15 model patterns differ by the time at which they reach their maximal values, so there is a direct relationship between clusters A to F and the temporal phases during metabolic cycle (Figure 4A). For instance, cluster A (262 genes) and B (77 genes) comprised genes whose EDPM decomposition preferentially followed model patterns n°1 and n°2, respectively. The mRNAs of these genes peak at the very beginning of the metabolic cycle (between 0 and 75 minutes). Genes in clusters C (123 genes) and D (27 genes) mainly conformed to model patterns n°5 and 8 and are expressed in the middle of the metabolic cycle (between 75 and 200 minutes). Finally, clusters E (30 genes) and F (107 genes) followed model patterns n°11 and 13 and comprised genes that are expressed at the end of the metabolic cycle (between 200 and 300 minutes).

#### Functional discrimination between mRNAs present during phases A to F

Previous cluster analysis of the entire microarray data set identified three superclusters of gene expression termed R/B (reductive, building), R/C (reductive, charging) and Ox (oxidative) [3]. Our analysis is consistent with this three cluster organization but it offers a more refined view of transcriptome dynamics. R/B genes are found in phases A and B, R/C genes in phases C and D, and Ox genes in phases E and F. To assess the biological relevance of the chronological order of the transcriptional classes A to F, we grouped the genes involved in mitochondria biogenesis into eleven model functional groups (see Dataset S4 for a detailed list of genes attributed to each functional group). Seven of these groups are shown in Figure 4B. They are labeled “Translation machinery” (83 genes), “Translation regulation” (47 genes), “Assembly factors” (32 genes), “Protein import” (40 genes), “Respiratory chain complex” (34 genes), “TCA cycle” (21 genes) and “Amino acid synthesis” (31 genes). We determined the percentage of genes in each biological function that belong to each temporal class A to F and there was a clear chronological distribution, highly biologically relevant (Figure 4B). For instance, functional discrimination between phases A and B is critical for respiratory chain complex assembly: almost all the genes (∼78%) encoding assembly factors for these complexes have a high mRNA level in phase A (see the function named “Assembly factors”), whereas most genes (∼56%) encoding structural units are present in phase B (function named “Respiratory chain complex”). This is important because temporal discrimination between the two gene classes probably facilitates complex construction: assembly factors being required at the start of subassembly intermediate formation (see, for instance, the case of Shy1p in COX assembly [16]). More generally, mRNAs coding for mitochondrial proteins peak at different times during the yeast metabolic cycle. The first mRNAs to appear are those for genes whose function is associated with the translation machinery (or regulation) and assembly factors (Figure 4B, phase A), followed by those involved in the synthesis of the respiratory chain structural proteins (Figure 4B, phase B) and finally mRNAs coding for enzymes involved in amino-acid biosynthesis are more abundant in phase F. This implies that mRNAs and probably the corresponding mitochondrial proteins are produced sequentially along the yeast metabolic cycle described by Tu *et al.* [3].

### Coupling and coordination of periodic gene expression: cross-talk between transcription and translation

#### Coordination between mRNA oscillations and translation site in the cytoplasm

We compared the cellular localization of translation of the mRNAs for genes in clusters A to F. We previously described three classes of nuclear mRNAs encoding mitochondrial proteins, differing in their sites of translation [10]. Class I and II mRNAs are found near mitochondria, whereas class III mRNAs are translated on free cytoplasmic polysomes. The subcellular localization of class I mRNAs is dependent on Puf3p, whereas class II mRNAs are Puf3p independent. The distribution of mRNAs of these three translation classes among the successive temporal phases (A to F) is represented in Figure 4C. The most salient feature is the substantial overlap between phase A and class I genes. Class I mRNAs dominate in phase A (183 of 262 genes), with only a few class I genes in other phases. Class II and III mRNAs are more evenly distributed, with the frequency of class II mRNAs being highest in phase C, and class III mRNAs being more frequently present in phases D, E and F. These observations imply coordination between mRNA oscillations and site of translation in the cytoplasm. This phenomenon is the consequence of transcriptional and post-transcriptional regulations and is presumably controlled by complex coordination of *trans*-acting factors acting on *cis* elements that remain to be identified. We therefore systematically searched for *cis*-acting signals in 5′ and 3′ UTR sequences, using several approaches (see below).

#### Identification of 5′ cis-regulatory elements

**Figure 5:**
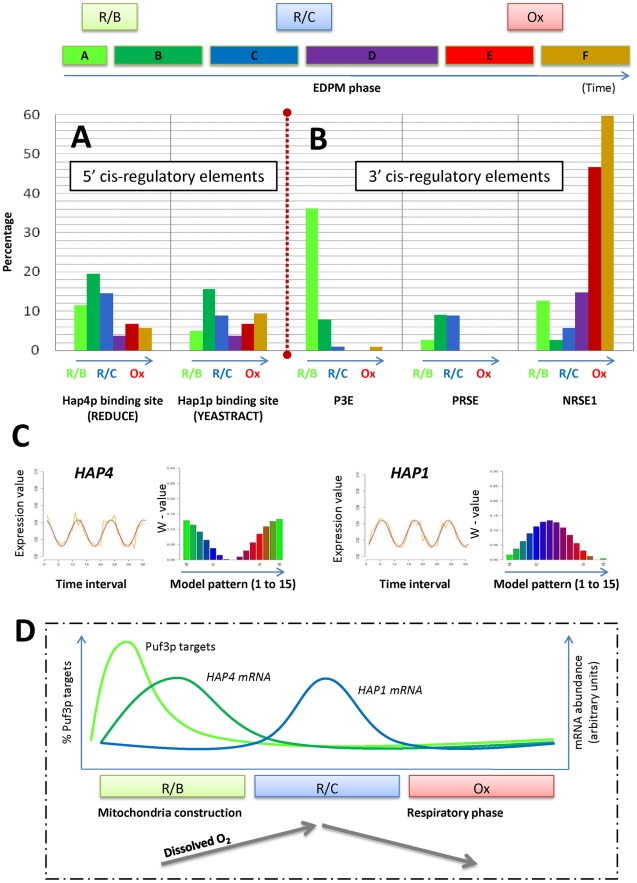
Cis- and trans-acting elements of the mitochondrial cycle: transcriptional and post-transcriptional regulations. The upper line shows the correspondence between the previously defined metabolic phases (R/B, R/C, Ox, [3]) and the phases A to F defined in this work as relevant to the 626 oscillating nuclear genes, which code for mitochondrial proteins. The colors of the bar reflect the corresponding phases of the metabolic cycle (green = R/B; blue = R/C; red/brown = Ox). **A** and **B** show the percentage of genes in each phases A to F that contain cis-acting regulatory motif in 5′ and 3′ UTR regions, respectively. The significant motifs were identified using various bioinformatic tools (MatrixREDUCE [11], YEASTRACT [14]) or from published motifs whose consensus sequences are P3E = CCUGUAAAUACCC, PRSE = UAUAUAUUCUUA, NRSE1 = UUUGAUAGACUC [12]. C: Oscillating concentrations of *HAP4* and *HAP1* mRNAs analyzed with EDPM. They were found to peak during phases A and C, respectively. *HAP1* mRNA peaks when the dissolved oxygen concentration is maximum (phase R/C of Tu *et al.* [3]). This is in agreement with observed oxygen-dependent transcription regulation of *HAP1* (see the main text). The only known trans-acting factor recognizing the 3′ motif P3E is Puf3p [10],[12],[17]. *PUF3* mRNA does not significantly oscillate and could not be precisely assigned to a particular phase of the mitochondrial cycle (data not shown). D: Schematic summary of the above data. The % of Puf3p target mRNAs and the abundance of the mRNAs coding for the two transcription factors Hap4p and Hap1p are represented along one oscillatory period. The *HAP4* mRNA variations coincide with the abundance of genes with a Hap4p binding site in their promoter and the *HAP1* mRNA follows the variations of dissolved O_2_. This is in agreement with the property of O_2_ sensor previously described for Hap1p (see text).

We investigated the regulatory processes governing the tight coordination of gene expression in phases A to F, by applying the MatrixREDUCE algorithm [11]. The motif with the highest score was CCAATCA (see Dataset S5 for complete results). This motif is compatible with the binding site of the transcription factor Hap4p [14], a transcriptional activator and global regulator of respiratory gene expression. The proportion of genes belonging to each A to F phases for which Hap4p binding sites were found in upstream sequences is presented in Figure 5A. Nineteen % of the genes in cluster B and 15% of the genes in cluster C contain Hap4p binding sites. The corresponding enrichment p-values are significant, at 3×10^−5^ (cluster B) and 2×10^−4^ (cluster C) (see Methods). We analyzed other *HAP* genes encoding transcription factors involved in the regulation of gene expression in response to oxygen levels. The *HAP1* gene was identified as particularly interesting because (*i*) its RNA level, like that of *HAP4*, varies substantially during the metabolic cycle (see Figure 5C) and (*ii*) the upstream sequences of more than 15% of the genes in cluster B (p-value = 7×10^−4^) have a Hap1p binding site [14]. Thus, the transcription factors Hap4p and Hap1p are excellent candidates to play an important role in the regulation of the yeast metabolic cycle.

#### Identification of 3′ cis-regulatory elements

Our search of regulatory elements in 3′ UTR sequences used work by Foat *et al.* [12], in which the authors used their MatrixREDUCE algorithm to identify binding sites for six mRNA stability regulators in *Saccharomyces cerevisiae*. The consensus sequences for these motifs are CCUGUAAAUACCC ( = P3E), UUAUGUAUCAUA ( = P4E), UAUAUAUUCUUA ( = PRSE), CUGAUUACACGG ( = RUPE), UUUGAUAGACUC ( = NRSE1), UUGUGUAAUCCAUCGAUCAU ( = NRSE2) and determine binding specificity for several RNA-binding proteins, including Puf3p (P3E motif) and Puf4p (P4E motif). We assessed the potential link between the occurrence of these motifs in 3′ UTR sequences and the temporal phases A to F by calculating the proportion of genes in each cluster with at least one motif. The P3E, PRSE and NRSE1 motifs appeared to be significantly overrepresented in phases A (p-value 6×10^−120^), B/C (6×10^−8^) and F (1×10^−9^), respectively (Figure 5B). The P3E motif is the site recognized by the RNA-binding protein Puf3p [12], which contributes to localizing mRNAs close to mitochondria [10]. We extended this observation, by considering all Puf3p targets experimentally determined by [17] (Text S2), and observed that more than 80% of the Puf3p mRNA targets are found in phase A. The presence of a Puf3p motif in class A genes is fully consistent with the translational properties of class I mRNAs: the localization of these mRNAs near mitochondria is altered when *PUF3* is deleted [10].

### Novel candidate genes involved in regulation of mitochondrial functions

**Figure 6:**
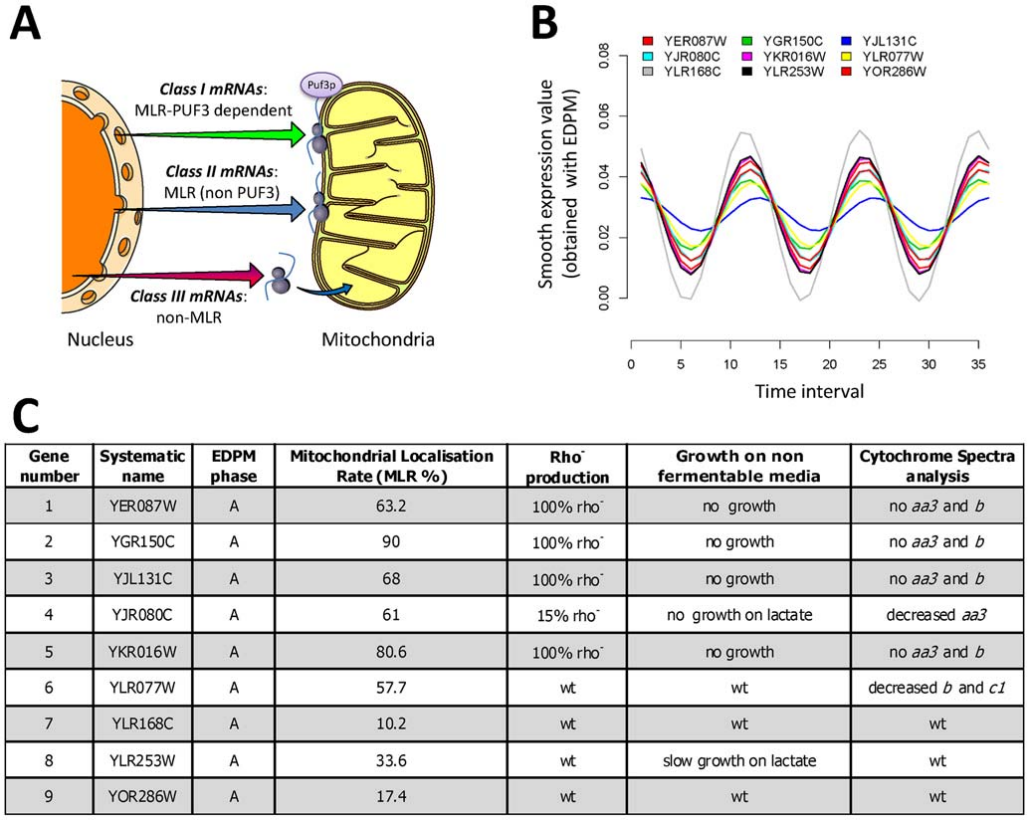
Experimental validation of the EDPM cluster analysis. This cluster analysis predicts that genes whose mRNAs peak in phase A and which are localized to mitochondria under the control of Puf3p (class I mRNAs, upper left, **A**) are likely to code for important elements of early steps of mitochondrial biogenesis. Nine completely uncharacterized genes were chosen on the basis of a perfect cluster A expression profile. ***M*** vectors for these nine genes, obtained by multiplying their EDPM vector ***W*** by the model pattern matrix ***P*** (see Figure 1) are represented in **B**, using a different color for each gene. C: Phenotypic analysis of the strains deleted for each of these nine genes is presented (Wt = wild type). Cytochromes *c_1_* and *b* are part of respiratory complex III, and cytochromes *aa_3_* are part of complex IV. Note that only two strains, YLR168C and YOR286W did not have altered respiratory properties.

This analysis leads to the prediction that unknown cluster A genes translated in the vicinity of mitochondria in a Puf3p-dependent way (class I) are likely to be involved in early steps of mitochondria biogenesis. To test this experimentally, we examined the properties of nine strains carrying deletions of uncharacterized cluster A/class I genes (Figure 6B and Dataset S3). For each mutant strain, we checked the ability to grow on non fermentable carbon sources and tested the assembly of respiratory complexes III and IV by recording cytochrome spectra (see Methods). Disturbance of early steps of mitochondrial biogenesis —for example replication of mitochondrial DNA, mitochondrial transcription and translation— can affect maintenance of mitochondrial DNA [18], we also tested whether these mutant strains retained the mitochondrial chromosome by measuring the production of petite cells (rho^−^). The phenotypes of these deleted strains are presented in Figure 6C. Strikingly, seven out of the nine gene-deleted strains displayed severe respiratory dysfunctions (poor growth on non-fermentable media) and/or alterations in their cytochrome spectra. These phenotypes strongly suggest that most of the unknown phase A/class I genes have functions in mitochondrial transcription/translation or assembly of respiratory complexes. This is strongly in favour of the idea that during this short period (phase A lasts only 25 minutes, Figure 4A), there is a surge in the abundance of mRNAs important for mitochondrial biogenesis and that they are translated at particular subcellular localization.

## Discussion

### Gene cluster analysis and dynamics of RNA regulons in mitochondrial biogenesis

It was recently observed [3],[19] that yeast cells can be synchronized and exhibit synchronous waves of storing and then burning carbohydrates. Using microarrays, it was shown that many nuclear genes coding for mitochondrial proteins, have their mRNAs which oscillate and peak at a time when highest rate of respiration has passed. It was suggested [19] that cells are either rebuilding or duplicating their mitochondria at this time. We took advantage of these data to better analyze the mitochondria rebuilding program and identified new gene clusters reflecting spatio-temporal groups of gene expression. Our findings are entirely consistent with the notion of RNA regulons [7],[8], according to which mRNA-binding proteins (RBP) play an important role, coordinating the various post-transcriptional events. We show here that 262 mRNAs coding for important mitochondrial proteins (assembly factors, ribosomal proteins, translation regulators) are coordinately and periodically present in increasing amounts early in the mitochondrial cycle (phase A = 25 minutes). In addition, most of these mRNAs are specifically localized in the vicinity of mitochondria under the control of the protein Puf3p. This suggests that during this particular time-window, Puf3p acts in the control of mRNA localization/translation. During the rest of the mitochondrial cycle, Puf3p may function (possibly in association with other RBPs) either in mRNA degradation [20] or in the control of bud-directed mitochondrial movement [21]. Following this early phase A, phases B (50 minutes) and C (50 minutes) concern elements of the fundamental mitochondrial machineries (respiratory chain complexes, TCA cycle, etc.). Undoubtedly, this chronology of events should reflect the logic of mitochondria construction.

### Phase A gene expression is a fundamental step in the mitochondrial cycle: the case of COX assembly

This point can be illustrated with the well-documented assembly process of cytochrome c oxidase (COX) [22],[23], a fascinating process involving the sequential and ordinate addition of 11 subunits to an initial seed consisting of Cox1p (Table 1, “core” and “shield proteins”). In addition to the structural subunits, a large number of accessory factors are required to build the holoenzyme. Unexpectedly, we found that all the mRNAs for these accessory factors are relatively abundant early in mitochondrial biogenesis, that is during phase A. Cluster A includes genes whose expression is essential for a preliminary step, consisting of the synthesis of all the elements (RNA polymerase, ribosomes, translation factors) required for mitochondrial production of Cox1p; this step is followed by the construction of the core enzyme (Cox1p+Cox2p+Cox3p). We also observed that the mRNAs coding for the 18 assembly factor transcripts involved in COX assembly [22],[24] are mostly found during phase A (Table 1, “assembly factors”) and, in addition, all but one are translated in the vicinity of mitochondria under the control of Puf3p (MLR class I, [10]). The situation is very different for structural COX proteins (shield proteins of the complex). Except for Cox5A, all the corresponding mRNAs are found in phase B, indicating that the corresponding genes are expressed after those of phase A. Unlike phase A mRNAs, they are all translated on free cytoplasmic polysomes (MLR class III, [10]). This scenario agrees with the previous biochemical description of short intermediates [23]; especially interesting is the observation that Cox5Ap, found here in phase A, was previously identified as the first structural protein added to the S2 complex [23]. The properties of COX assembly described here are common to the other respiratory chain complexes. The mRNAs for assembly factors mostly peak in phase A and they are translated close to mitochondria, under the control of Puf3p; they initiate the formation of respiratory complexes by the successive addition of structural proteins whose mRNAs mostly peak in phase B. This is the first evidence that, at least in the conditions described in [3], the construction of the respiratory chain is one of the first steps of mitochondrial biogenesis; indeed, all the production machinery (assembly factors, translation, etc.) are available in phase A to produce and assemble the protein complexes in phase B.

**Table 1:** COX assembly and the mitochondrial cycle.

	ORF	Gene name	Functional class	MLR = % of mRNA associated with mitochondria	Presence of Puf3p binding site in 3′UTR	MLR class	Expression phase (This work)
**ASSEMBLY FACTORS**	YBR024W	SCO2	RCCasm4	73.7	Yes	I	B
	YBR037C	SCO1	RCCasm4	72.7	Yes	I	A
	YDL107W	MSS2	RCCasm4	22.5	Yes	I	A
	YDR079W	PET100	RCCasm4	15.4	Yes	I	A
	YDR231C	COX20	RCCasm4	17.8	Yes	I	C
	YDR316W	OMS1	RCCasm4*	100.0	Yes	I	A
	YER058W	PET117	RCCasm4	43.1	Yes	I	A
	YER154W	OXA1	RCCasm4*	67.4	Yes	I	A
	YGR062C	COX18	RCCasm4	45.5	Yes	I	F
	YGR112W	SHY1	RCCasm4	77.1	Yes	I	A
	YHR116W	COX23	RCCasm4	37.7	Yes	I	A
	YIL157C	COA1	RCCasm4	26.7	Yes	I	A
	YJR034W	PET191	RCCasm4	19.1	Yes	I	A
	YLL009C	COX17	RCCasm4	30.9	Yes	I	A
	YLR204W	COX24	RCCasm4	13	Yes	I	/
	YML129C	COX14	RCCasm4	22.6	Yes	I	A
	YOR266W	PNT1	RCCasm4	33.6	Yes	I	A
	YPL132W	COX11	RCCasm4	59.2	Yes	I	A
	YPL172C	COX10	RCCasm4	93.4	Yes	I	A
	YJL003W	COX16	RCCasm4	0	Yes	III	/
**SHIELD PROTEINS**	YDL067C	COX9	RCC-IV	0	No	III	/
	YGL187C	COX4	RCC-IV	0	No	III	B
	YGL191W	COX13	RCC-IV	0	No	III	B
	YHR051W	COX6	RCC-IV	0	No	III	B
	YIL111W	COX5B	RCC-IV	0	No	III	/
	YLR038C	COX12	RCC-IV	0	No	III	B
	YLR395C	COX8	RCC-IV	0	Yes	III	B
	YMR256C	COX7	RCC-IV	0	No	III	B
	YNL052W	COX5A	RCC-IV	0	No	III	A
**CORE PROTEINS (MITO-ENCODED)**	Q0045	COX1	RCC-IV	100.0	No	IV	No data in Tu *et al.*
	Q0250	COX2	RCC-IV	100.0	No	IV	No data in Tu et al.
	Q0275	COX3	RCC-IV	100.0	No	IV	No data in Tu et al.

COX (cytochrome c oxidase) assembly is a highly regulated multi-step process involving discrete short-term intermediates, S1, S2, S3 [23]. The table describes the known components involved in COX assembly and the relevant properties of the corresponding mRNAs. MLR (Mitochondria Localization of nuclear-encoded mRNA) characteristics are from [10]; classes indicate whether the mRNAs are translated on Puf3p-dependent (class I), mitochondria-linked (class II) or on free polysomes (class III). Phases A to C correspond to the early time-window of the mitochondrial cycle. The first step of COX assembly is the site-specific translation of the mitochondrially encoded COX subunits. For instance, COX1 mRNA is translated under the control of the translation regulators MSS51 and PET309 (both are class I mRNAs present during phase A, Dataset S3). The second step is the addition of Cox5p and Cox6p; note that Cox5p is the only structural subunit belonging to phase A, consistent with its role in early assembly step [25]. The last step is the addition of the rest of the nuclear-encoded subunits (shield proteins). These two last steps require the presence of the assembly factors. Note that most of the assembly factor transcripts appear during phase A, whereas the shield protein transcripts are present during phase B. In addition, assembly factor transcripts are localized to the vicinity of mitochondria (MLR, class I) and depend on Puf3p for this localization. Shield protein transcripts are translated on free polysomes (MLR class III) and have no Puf3p binding site.

### Transcriptional and post-transcriptional regulations alternate through the mitochondrial cycle

Genes coding for mitochondrial proteins can be classified into two different regulatory systems. This dichotomy is well illustrated in the case of OXPHOS complexes coding genes. The first class corresponds to mRNAs translated to the vicinity of mitochondria, mainly present in phase A and which code, for instance, for assembly factors. Genes of the second class code for structural proteins, and are found mainly in phases B or C during which transcription regulation is the major mechanism. Previous studies suggested that genes coding for assembly factors are not transcriptionally regulated [25]. We confirmed and extended these preliminary observations by showing that genes encoding assembly factors: (*i*) are expressed before genes encoding structural proteins, (*ii*) have a functional Puf3p binding site which controls localization/translation to the vicinity of mitochondria and may thus generate discrete foci on the matrix face of the mitochondrial membrane, and (*iii*) do not contain any evident signals in their 5′UTR, a feature which distinguishes them from the genes encoding structural proteins. The mRNAs for translation and assembly factors are all expressed only during phase A, but mRNAs for structural proteins are found during phases A, B and to a lesser extent C. This is likely to reflect the timing of the building of the various complexes. Thus, for instance, COX assembly requires an intact functional ATPase [22], which is in agreement with the fact that mRNAs for ATPase structural proteins are mostly found in phase A (see Dataset S4) whereas the COX equivalents are mostly in phase B (see Dataset S4). Also, unlike genes encoding assembly factors, genes coding for structural proteins of the respiratory chain complexes are mainly controlled transcriptionally. According to the environmental conditions, either Hap4p (depending on carbon availability [26],[27]) or Hap1p (depending on oxygen concentration [28]), regulate the transcription of nuclear genes coding for structural proteins. Binding sites for these two transcription factors are present significantly more frequently than expected from a random distribution in the genes of clusters A, B and C (Figure 5A). In addition, the amounts for both *HAP4* mRNA and *HAP1* mRNA also oscillate and peak in phases A and C, respectively (Figure 5C). *HAP1* mRNA variation is interesting because Hap1p can repress its own transcription and may act either as a repressor or as an activator, depending on oxygen levels [29]. It was observed that fluctuating levels of O_2_ dissolved in the culture, indicates changing activities of mitochondrial oxygen consumption and cellular redox switching [30]. Thus, Hap1p, which is an oscillating redox sensor, is an excellent candidate to signal the transition between non-respiratory rebuilding and respiratory phases (Figure 5D).

Overall, we report a comprehensive picture of the biogenesis of yeast mitochondria and illustrate spatio-temporal differences between groups of nuclear genes. The unexpected finding that transcriptionally or post-transcriptionaly regulated groups of genes are expressed both at different times and translated in different places may be of relevance to mitochondria in other species. Indeed, mammalian β F_1_-ATPase mRNA is found in the outer membrane and is translated, under the control of 3′UTR signals and RNA-binding proteins [31], only during cell cycle phase G2/M [32]; this gives credence to the general applicability of our observations. Studies with human cells are currently underway to assess the similarities and differences between yeast and human cells regarding these aspects of mitochondrial biogenesis.

## Supporting Information

Figure S1 Figure that describes the principle of the EDPM procedure.
(0.38 MB PDF)

Text S1 Detailed justifications for the use of EDPM algorithm together with several methodological controls.
(1.60 MB PDF)

Text S2 Distribution of all Puf3p targets (as determined experimentally by Gerber et al.) across the A to F phases.
(0.43 MB PDF)

Dataset S1 Complete gene lists with expression measurements from Tu et al. [2], for the 626 genes analyzed in this study.
(0.46 MB XLS)

Dataset S2 EDPM results for each of the 626 genes analyzed in this study.
(0.20 MB XLS)

Dataset S3 Distribution of genes into the six temporal phases of the mitochondrial cycle.
(0.21 MB XLS)

Dataset S4 Distribution of genes into 11 major functional classes, and properties of the genes.
(0.12 MB XLS)

Dataset S5 Detailed results obtained with the MatrixREDUCE algorithm, searching for 5′ regulatory signals.
(0.02 MB XLS)
