# Supplementary material for: Spatio-Temporal Dynamics of Yeast Mitochondrial Biogenesis: Transcriptional and Post-Transcriptional mRNA Oscillatory Modules
Source: PLoS Comput Biol. 2009 Jun 12;5(6):e1000409. doi: 10.1371/journal.pcbi.1000409 (PMC2690403; doi:10.1371/journal.pcbi.1000409)
Supplement: Figure S1 — Figure that describes the principle of the EDPM procedure. (0.38 MB PDF) [file pcbi.1000409.s001.pdf]

Figure S1: Illustration of the EDPM procedure

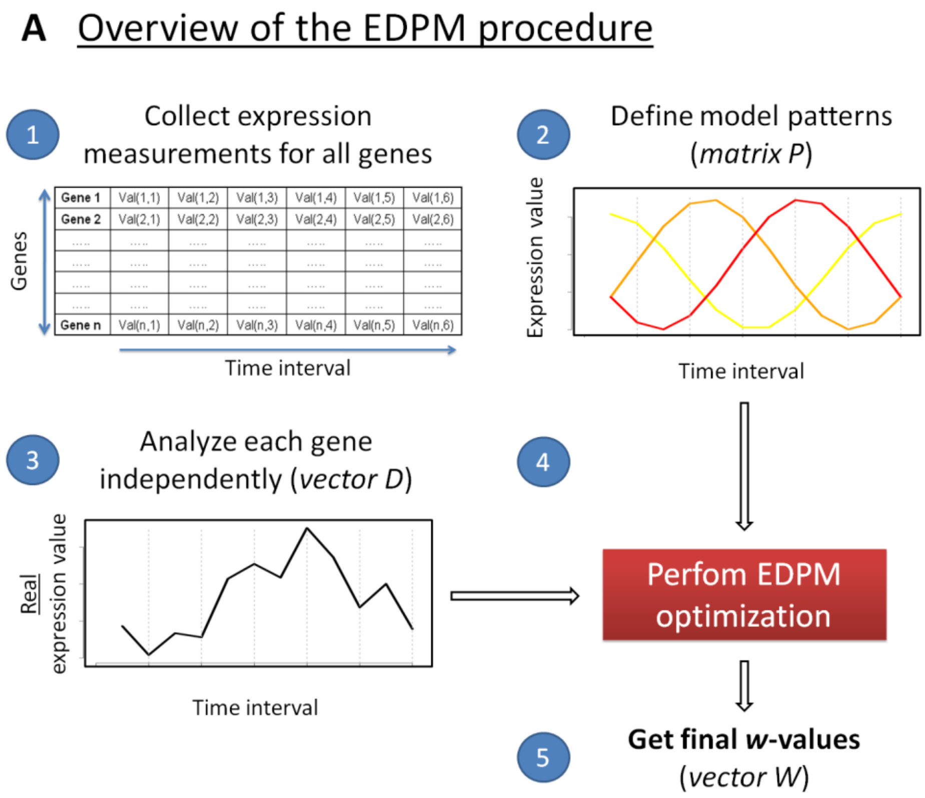

Figure S1: Overview of the EDPM procedure. *The EDPM procedure used in this analysis of mitochondrial genes can be decomposed into 5 major steps. (1) The microarray measurements for all genes to be analyzed are collected. (2) Model patterns are defined in order to match specific properties shared by the gene expression patterns collected in (1). In this illustration, 3 model patterns are represented (colored respectively in yellow, orange and red). They are oscillatory functions (one cycle), with constant period but different phases. They correspond to the matrix P presented* ***Figure 1A*** *(main text). (3) The EDPM analysis is performed analyzing expression measurements for one gene at a time. This vector corresponds to the vector D presented* ***Figure 1A*** *(main text). (4) EDPM calculates a vector W of w-values. Note that illustration of the EDPM optimization is presented* ***Figure 2*** *(main text). (5) The final w-values indicate the contribution of each model pattern to the expression pattern observed for the gene shown in (3).*
