# Supplementary material for: Spatio-Temporal Dynamics of Yeast Mitochondrial Biogenesis: Transcriptional and Post-Transcriptional mRNA Oscillatory Modules
Source: PLoS Comput Biol. 2009 Jun 12;5(6):e1000409. doi: 10.1371/journal.pcbi.1000409 (PMC2690403; doi:10.1371/journal.pcbi.1000409)
Supplement: Text S1 — Detailed justifications for the use of EDPM algorithm together with several methodological controls. (1.60 MB PDF) [file pcbi.1000409.s002.pdf]

Text S1: The EDPM methodology is well-adapted to the study of the gene subset coding for mitochondrially localized proteins

1) Why EDPM?

In their original study, Tu *et al.* (Tu, Kudlicki et al. 2005) performed an unbiased *k*-means cluster analysis of the entire microarray dataset and classified the oscillating genes into three major groups, called R/B (977 genes), R/C (1 510 genes) and Ox (1 023 genes). They demonstrated that these clusters were enriched in functionally related genes. In particular, nuclear-encoded mitochondrial genes were mostly founded in the R/B cluster that comprises genes whose mRNA levels peak when cell begin to cease oxygen consumption.

In this study, our aim was to better characterize the behavior of the genes involved in the mitochondrial biogenesis, during the Yeast Metabolic Cycle (YMC). Unlike previous studies like (Palumbo, Farina et al. 2008), we decided to focus our analysis on a subset of 626 genes that *(i)* are nuclear genes known to encode proteins found in mitochondria (Saint-Georges, Garcia et al. 2008) and *(ii)* were identified by (Tu, Kudlicki et al. 2005) as being expressed in a periodic manner.

Classical clustering methods (like hierarchical clustering or k-means) based on pair-wise correlations or distance calculations between expression measurements may yield many biological insights, but they were not optimal for analyzing temporal gene expression datasets. They make the implicit assumption that the data at each time point are collected independently of each other, thus ignoring the sequential nature of temporal gene expression data. In this respect, the approach proposed by Moloshok *et al.* (Moloshok, Klevecz et al. 2002) is particularly interesting since the authors were able to analyze cell-cycle data and order genes according to the cycle phases (G1, S, G2 or M). Our EDPM approach, by choosing in advance the model patterns used to decompose the expression profiles, is a simplified version of the method proposed by (Moloshok, Klevecz et al. 2002) in which aims at evaluating simultaneously the models patterns and the vector of *w*-values, and hence considerably increases the number of parameters to be estimated.

Our simplification has a major advantage specially when analyzing the gene expression patterns with specific properties, like Tu *et al.* dataset: periodic signals among 3 successive cycles and a unique period for all genes. On the other hand, this simplified approach reduces the number of microarray datasets to which EDPM can be (at the present time) successfully applied. However, even if the generalization of EDPM can be planned in the future, it is clearly outside the scope of this study restricted to a limited set of genes and the Moloshok *et al*. approach remains more general.

Several relevant properties of the EDPM approach to analyze the 626 mitochondrial gene expression profiles can be examined (see below): 2) evaluation of the optimized EDPM criterion with random sample datasets; 3) influence of the number of model patterns in the phase definition (A t F); and 4) comparative analysis of the clustering results obtained with EDPM *w*-values and other methodologies.

2) Evaluation of the optimized EDPM criterion with random sample datasets

The EDPM approach consists in calculated the ***W*** vector in order to minimize the square of the distance between the ***M*** and ***D*** vectors (see the main text for more details). For a given gene *i*, the criterion to be optimized — *i.e.* numerically minimized — to find the optimum solution of *w*-values is as follows:

$$S^i = \sum_{t=1}^{T}\left(a_t^i - \left(b\sum_{k=1}^{K}\omega_k^i m_{k,t} + c\right)\right)^2 + P^i$$

Where, a_t^i is the microarray expression measurement of gene *i* at time *t*, $m_{k,t}$ is the value of model pattern *k* at time *t*, K is the number of model patterns, *b* and *c* are parameters to adjust amplitude and expression level, and finally P^i is a penalty function to ensure that the sum of *w*-values is equal to 1.

In an ideal case, we would like the final S^i value (after the optimization procedure) to equal 0. In reality, the final S^i value mainly depends on the possibility to fit the real expression data with the proposed model patterns. The more the model patterns are adaptable to the observed gene expression measurements, the more the EDPM optimization is efficient, *i.e.* the final S^i value is close to 0.

To verify this idea and assess the significance of the final values obtained for the optimized EDPM criterion S^i, the expression measurements of the 626 mitochondrial genes were randomized by shuffling the values, and the EDPM decomposition was performed. Distributions of the final S^i values obtained for the 626 genes, using either real expression data or random expression data were compared **Figure S1.1** (below). As expected, we could observe that the EDPM criterion was significantly smaller using the real expression data than it was using the sample data. Such an observation justified the use of EDPM for the 626 genes analyzed in this study. They exhibit periodic gene expression profiles during the YMC (this was demonstrated by Tu *et al.*) and hence are compatible with the 15 model patterns used here (**Figure 1B**, main text). Moreover, it should be noted that the final *w*-values distribution is also a good indicator of the EDPM relevance. In case of shuffle data, the *w*-values are homogeneously distributed, indicating that no particular model pattern can explain the random expression profiles (see **Figure S1.2** for an illustration).

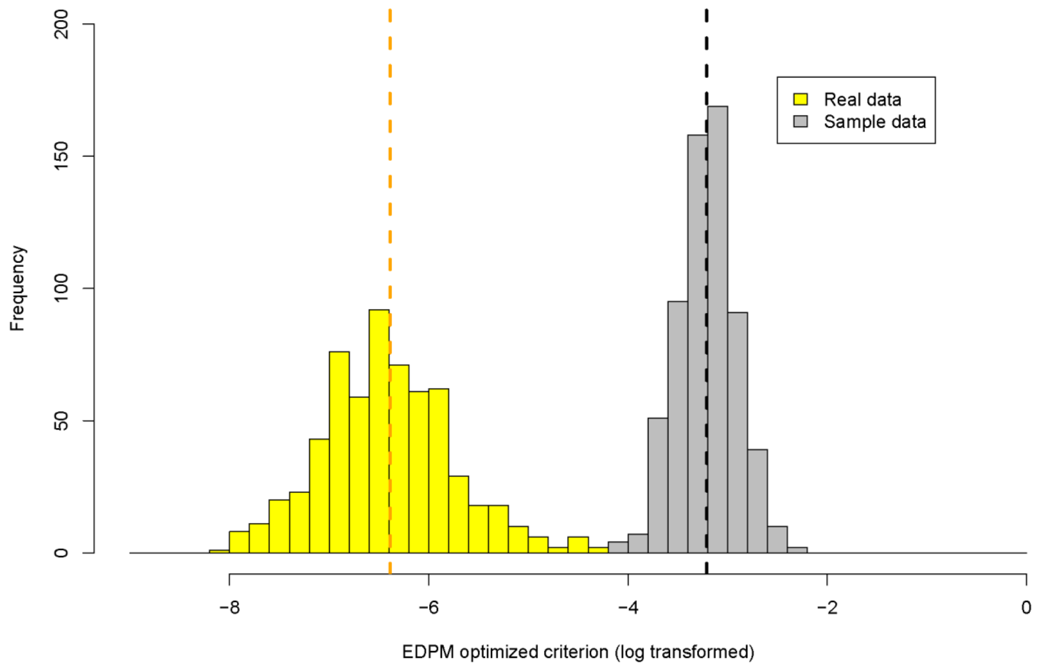

Figure S1.1: Histograms of final values of the EDPM optimized criterion for real and random sample datasets. *The EDPM optimization was performed using the real gene expression measurements of the 626 mitochondrial genes and a sample dataset obtained by shuffling the data. Final S^i values are shown here (yellow = real data; grey = sample data) after logarithm transformation to better visualize small variations. Mean of each distribution are indicated with dashed lines. Values obtained with real data are systematically lower than values obtained with sample data.*

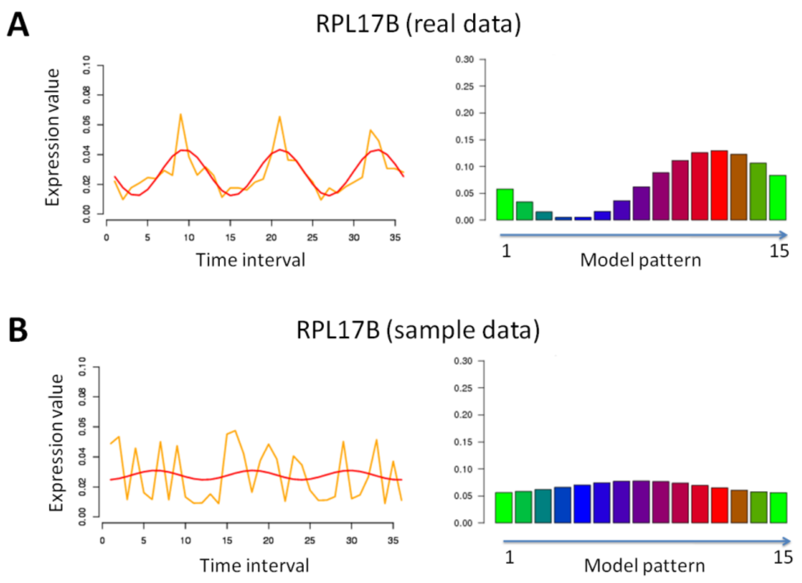

Figure S1.2: Comparison of EDPM results obtained with real expression data and random sample data. *As an illustration, gene RPL17B was analyzed using EDPM. Results obtained with the real expression measurements are shown in (A), and results obtained with shuffle data are shown in (B). In case of shuffle data, the w-values are homogeneously distributed, meaning that no particular model pattern can explain the random expression profiles.*

3) Influence of the number of model patterns on the phase definition (A to F)

The phases A to F presented in the main text were obtained using 15 model patterns in the EDPM procedure. To quantify the impact of this choice on the final repartition of the 626 mitochondrial genes into these 6 phases, the complete analysis (EDPM decomposition followed by a hierarchical clustering using *w*-values) was repeated varying the number of model patterns. Using as a reference the gene repartition into phases A to F presented in the main text (**Figure 3**), we calculated the percentage of genes that were "correctly classified", *i.e.* genes that kept the same phase assignment (for instance a gene that was classified in phase A using 15 model patterns is still classified in phase A using *n* model patterns). Results obtained with 3, 5, 7, 9, 11, 12, 13, 15, 17 and 19 model patterns are shown in **Figure S1.3** (below). We could observed that a minimal number of 9 model patterns is required to stabilize the gene repartition into phases A to F. Lower, the number of model patterns was not enough to precisely indentified the phase transitions. Between 9 and 19 model patterns, variations could be observe, but still the main phase organization was respected with around 80% of the genes that keep their original assignment.

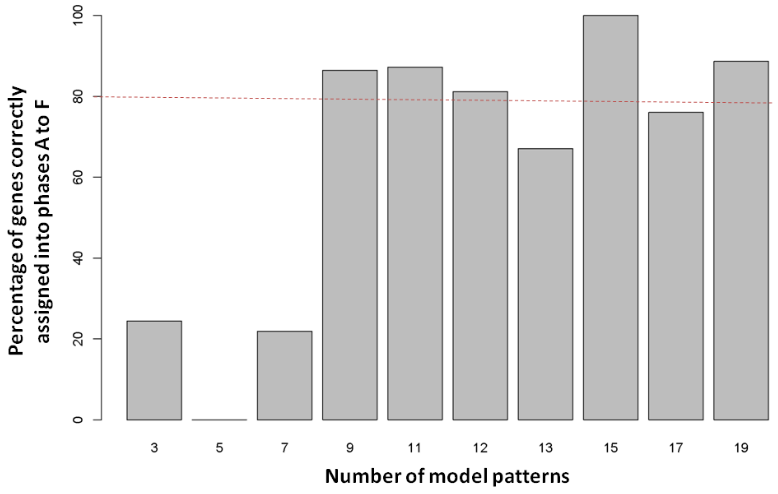

Figure S1.3: Effect of the number of EDPM model patterns in the final repartition of the 626 mitochondrial genes into the phases A to F. *The analysis of the 626 mitochondrial genes presented in Figure 3 (main text) was repeated varying the number of model patterns between 3 and 19. The percentage of genes "correctly assigned" was calculated using as a reference the gene repartition into phases A to F obtained in the main text. Around 80% (see the red dashed line) of the genes are correctly assigned if the number of model patterns is higher than 7.*

4) Comparative analysis of the clustering results obtained with EDPM *w*-values and others methodologies

a) Hierarchical clustering using original expression values

In the main text, the 626 genes involved in mitochondria biogenesis were analyzed with EDPM and classified in several clusters (also referred as "phases") according to their *w*-values. These clusters were named A to F and comprised a distinct subclass of genes whose mRNA levels is peaking in different time windows of the metabolic cycle (**Figure 3** and **Figure S1.4** below). The reality of clusters A to F is supported by the fact that most of the genes in each cluster share functional similarities (**Figure 4**). This is especially convincing for phase A.

Additionally, to verify that EDPM really improved the dissection of expression temporal waves, we carried out a new hierarchical clustering using the original expression values instead of the *w*-values derived from EDPM (same clustering algorithm and correlation measure). Six clusters (named A' to F') were thus obtained and temporally ordered according to the time point for which the mean expression profile reached its maximal value during the first metabolic cycle (**Figure S1.5**, below). Major differences between the two clustering approaches could be observed (see **Table S1.1**) and only 259 genes (41%) were classified in the same clusters (A and A'; B and B'; …; F and F').

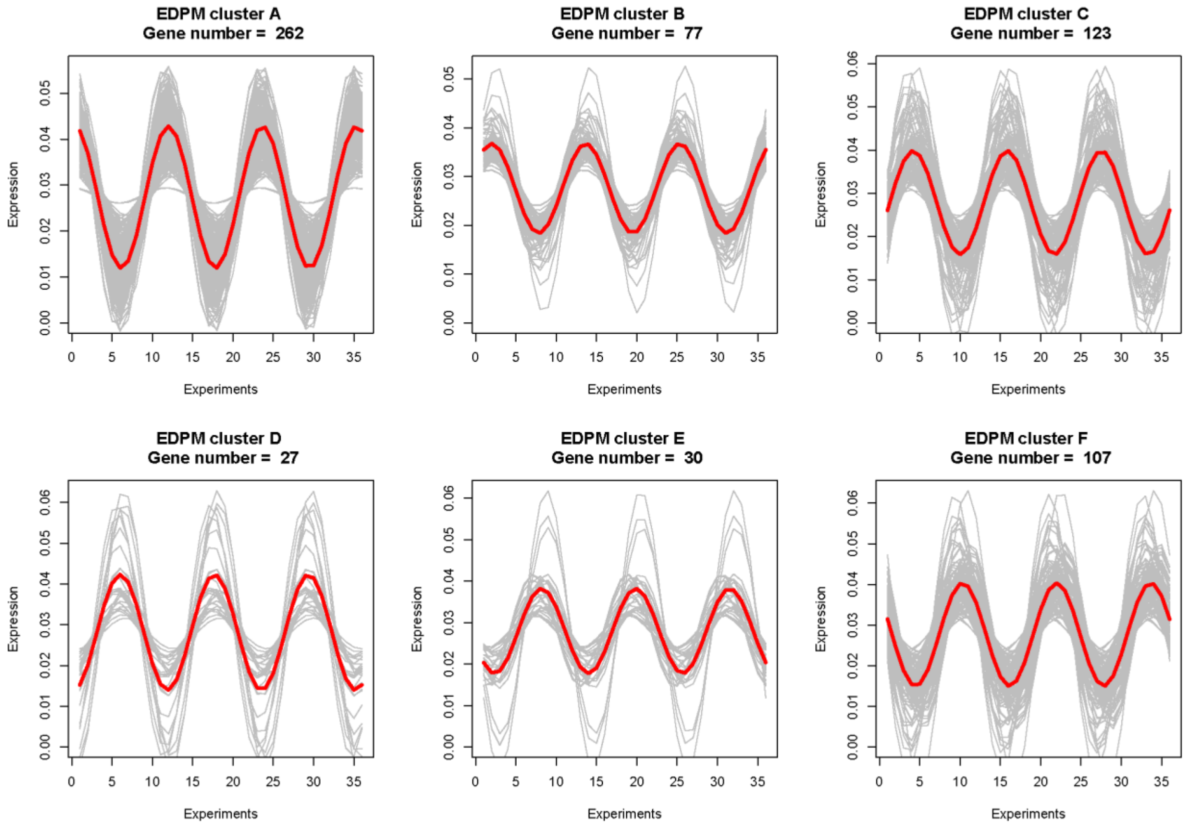

Figure S1.4: Hierarchical clustering using EDPM w-values. *Genes comprised in the clusters A to F presented in the main text are shown here. Grey lines correspond to their "EDPM expression profiles", the M vectors obtained by multiplying there w-values vectors by the model pattern matrix P (see Methods in the main text). Mean profile is represented in red.*

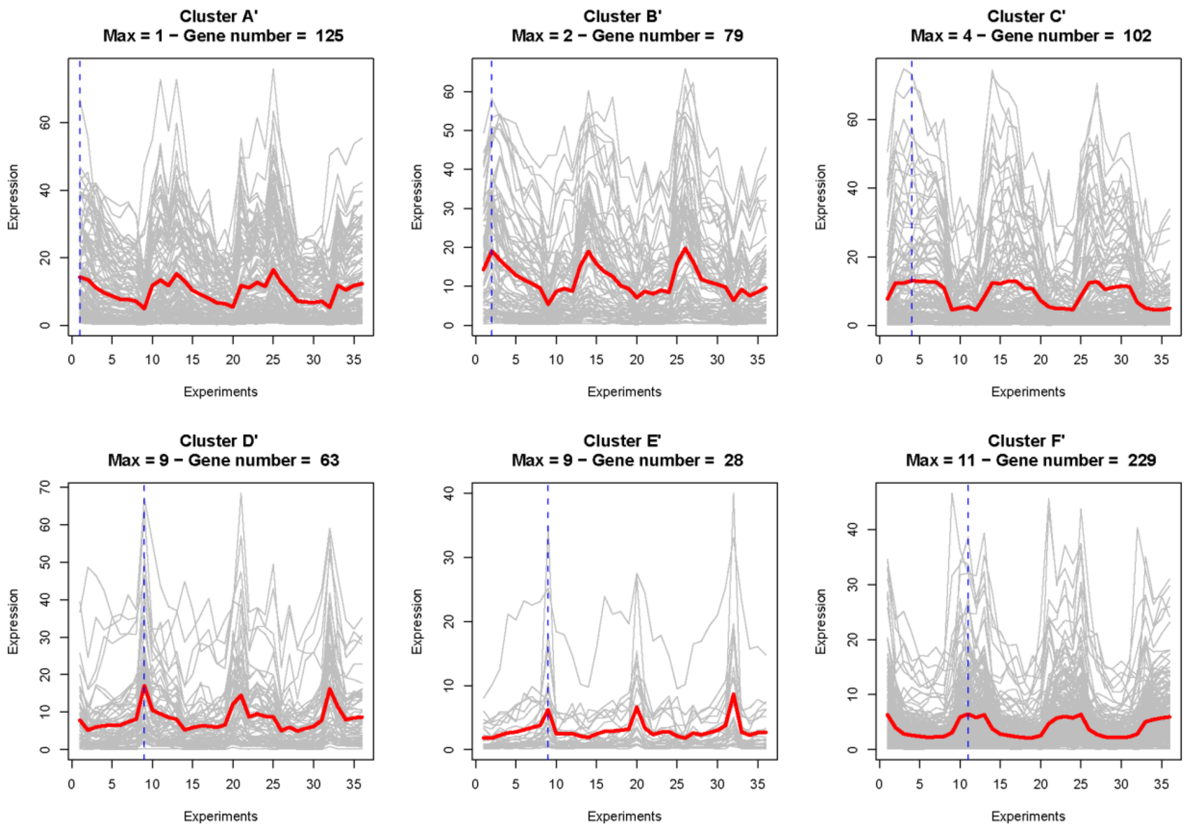

Figure S1.5: Hierarchical clustering using original expression values*. The 626 mitochondrial genes were classified according to the correlation measure between their expression profiles, using hierarchical clustering algorithm ("hclust" function available in R programming language, with Pearson correlation distance and the "ward" method for gene agglomeration). Six clusters (named A', B', C', D', E' and F') were obtained. Expression profiles of genes comprised in each cluster are shown here (grey lines), with the mean profiles in red. The clusters were ordered according to the time measurement for which the mean profile reaches its maximal value during the first cycle, i.e. between experiments 1 and 12 (indicated with a blue dashed line).*

		Hierarchical clustering using original expression values					
		A'	B'	C'	D'	E'	F'
Hierarchical clustering using EDPM w-values	A	80	0	0	2	0	180
	B	45	31	0	0	0	1
	C	0	48	75	0	0	0
	D	0	0	27	0	0	0
	E	0	0	0	5	25	0
	F	0	0	0	56	3	48

Table S1.1: Comparison of clustering results obtained with EDPM w-values and original expression values. *The same clustering algorithm was used to classify the 626 mitochondrial genes (hierarchical clustering, Pearson correlation distance and ward method for gene agglomeration), using either the w-values (clusters A-F in line) or the original expression values (clusters A'-F' in column). Expression profiles of genes in each cluster are shown in Figure S1.4 and S1.5. The gene repartition in each cluster is shown here. Only 259 genes are identically classified between clusters A-F and A'-F', they are indicated with red boxes.*

b) Evaluation of the biological relevance of the clusters A-F and A'-F': the case study of the COX assembly process

In the main text, we assessed the biological relevance of the chronological order of the transcriptional clusters A to F and found the separation of genes into clusters A and B especially interesting. In particular, the functional discrimination between these two clusters was critical for the assembly of cytochrome c oxidase (COX) (**Table 1**). Indeed, this biological process requires the sequential and ordinate expression of two types of genes: *(i)* genes coding for the COX assembly factors (18 genes, see **Table 1**) and *(ii)* genes coding for the shields proteins of the complex (7 genes, see **Table 1**). The discrimination of these two different classes of genes in different clusters is therefore a good indicator of the clustering approach. Below, it will serve as a reference to compare EDPM performances with others methodologies.

Each of the 25 genes involved in the COX assembly were thus extracted from the clusters A – F and A' – F' and in each cluster, we calculated the percentage of "assembly factor" and "shield protein" encoding genes. Results obtained with clusters A – F and clusters A' – F' are shown respectively **Figures S1.6** and **S1.7**. Clearly, the discrimination between genes involved in COX assembly is improved with EDPM. The COX assembly factors were mostly found in the cluster A (15/18), whereas genes coding for shield proteins were found in the cluster B (6/7).

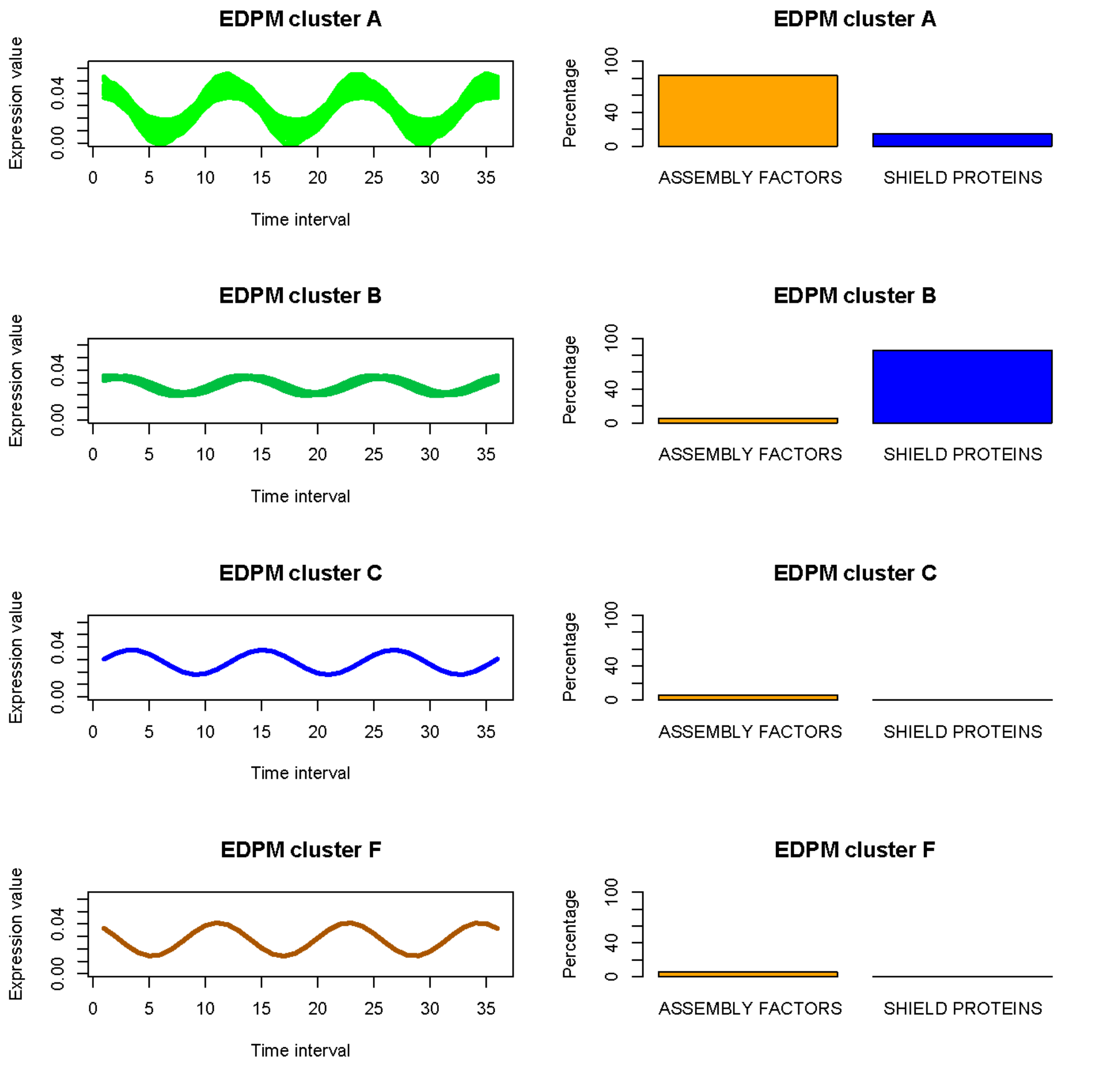

Figure S1.6: Functional discrimination between genes involved the COX assembly process (clustering with w-values). *From the 626 mitochondrial genes analyzed in this study, 25 are involved in the COX assembly process (see Table 1 and Table S1.2 for a complete list). There EDPM expression profiles are represented here (left panel) with a different color depending on the cluster A – F they belong to. For each cluster, the percentages of COX genes annotated as "assembly factors" or "shield proteins" are presented (right panel). The COX assembly factors are mostly found in the cluster A (15/18), whereas genes coding for shield proteins are found in the cluster B (6/7).*

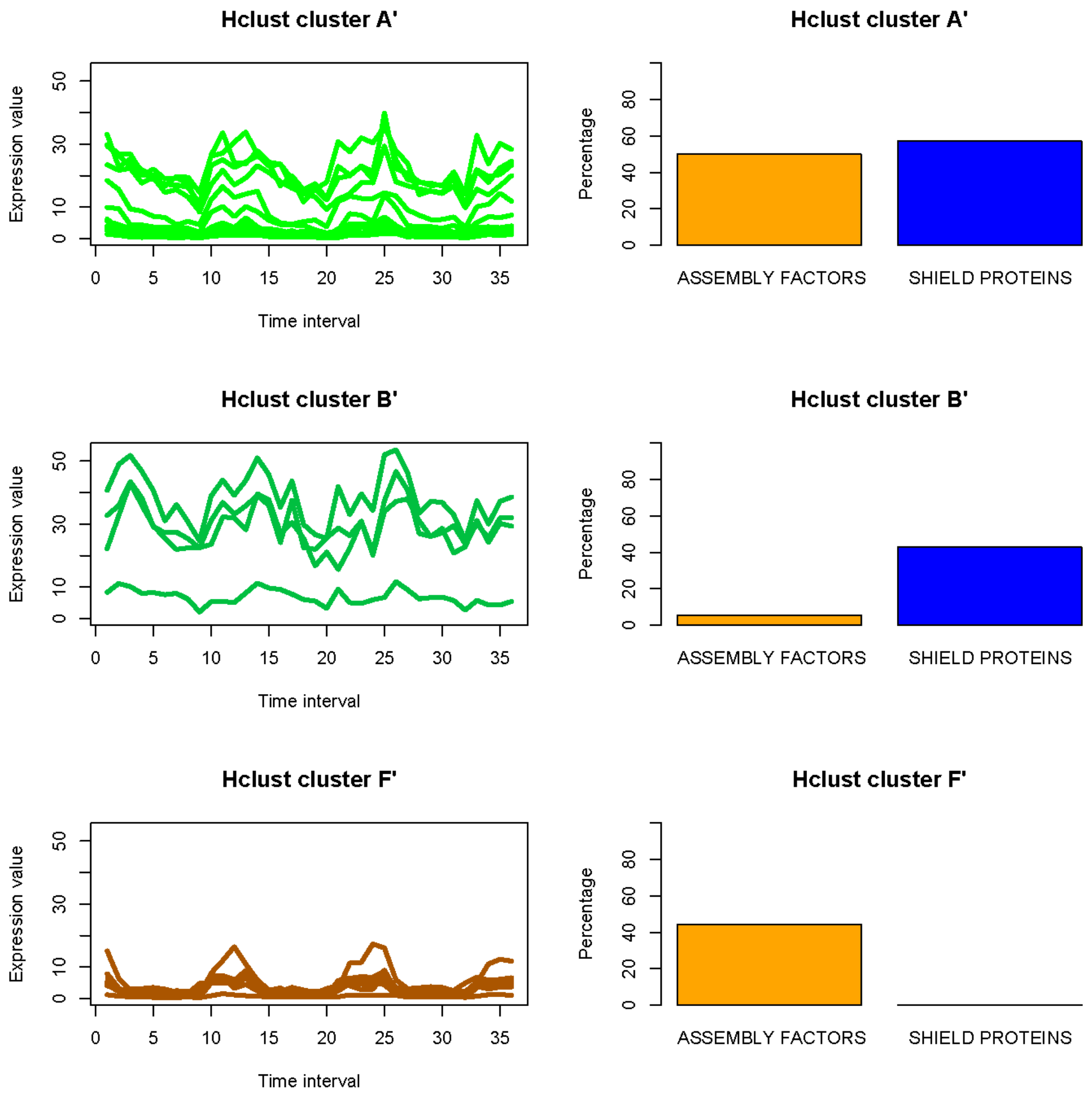

Figure S1.7: Functional discrimination between genes involved the COX assembly process (clustering with original expression data). *The analysis described Figure S1.6 was repeated, using the clusters A' – F' obtained using original expression data. Contrary to EDPM analysis (Figure S1.6), the discrimination between genes encoding COX "assembly factors" and "shield proteins" is only partial. In particular, the cluster A' comprised both type of genes in a significant proportion (9/18 assembly factors and 4/7 shield proteins).*

c) Comparison with other clustering methodologies

Previous results showed that EDPM improves the dissection of expression temporal waves (mostly as far as clusters A and B are concerned), compared to direct hierarchical clustering using original expression data. To go further in this observation, we tried to apply two other clustering algorithms. First, we chose the k-means algorithm because a simplified version of this algorithm using preselected "sentinel genes" was used by Tu *et al.* (Tu, Kudlicki et al. 2005) and Palumbo *et al.*

(Palumbo, Farina et al. 2008) in their previous analyses of YMC. Second, we chose the PAM algorithm (Partitioning of the data into k clusters "around medoids") that known to be a more robust version of k-means (see http://cran.r-project.org/web/packages/pamr/index.html for a detailed documentation). In both cases, Pearson correlation distance was used to quantify similarities between genes expression profiles. Six clusters were obtained and temporally ordered according to the time point for which the mean expression profile reaches its maximal value during the first metabolic cycle. Finally, we analyzed the discrimination between genes involved in COX assembly. The repartition of the COX genes into the obtained clusters (also named A' to F') is presented **Table S1.2** (below). Again, only EDPM allowed a clear discrimination between "assembly factors" and "shields proteins".

	ORF	Gene name	Functional class	MLR = % of mRNA associated with mitochondria	Presence of Puf3p binding site in 3'UTR	MLR class	EDPM expression phase (This work)	Hclust	K-means	PAM
ASSEMBLY FACTORS	YBR024W	SCO2	RCCasm4	73.7	Yes	I	B	A'	B'	C'
	YBR037C	SCO1	RCCasm4	72.7	Yes	I	A	A'	A'	A'
	YDL107W	MSS2	RCCasm4	22.5	Yes	I	A	A'	C'	D'
	YDR079W	PET100	RCCasm4	15.4	Yes	I	A	F'	A'	A'
	YDR231C	COX20	RCCasm4	17.8	Yes	I	C	B'	B'	C'
	YDR316W	OMS1	RCCasm4*	100,0	Yes	I	A	F'	A'	A'
	YER058W	PET117	RCCasm4	43.1	Yes	I	A	F'	A'	A'
	YER154W	OXA1	RCCasm4*	67.4	Yes	I	A	F'	A'	A'
	YGR062C	COX18	RCCasm4	45.5	Yes	I	F	F'	A'	A'
	YGR112W	SHY1	RCCasm4	77.1	Yes	I	A	A'	C'	D'
	YHR116W	COX23	RCCasm4	37.7	Yes	I	A	A'	C'	D'
	YIL157C	COA1	RCCasm4	26.7	Yes	I	A	A'	C'	D'
	YJR034W	PET191	RCCasm4	19.1	Yes	I	A	A'	C'	D'
	YLL009C	COX17	RCCasm4	30.9	Yes	I	A	F'	A'	A'
	YLR204W	COX24	RCCasm4	13	Yes	I	/	/	/	/
	YML129C	COX14	RCCasm4	22.6	Yes	I	A	A'	C'	D'
	YOR266W	PNT1	RCCasm4	33.6	Yes	I	A	F'	A'	A'
	YPL132W	COX11	RCCasm4	59.2	Yes	I	A	A'	A'	A'
	YPL172C	COX10	RCCasm4	93.4	Yes	I	A	F'	A'	A'
	YJL003W	COX16	RCCasm4	0	Yes	III	/	/	/	/
SHIELD PROTEINS	YDL067C	COX9	RCC-IV	0	No	III	/	/	/	/
	YGL187C	COX4	RCC-IV	0	No	III	B	A'	C'	D'
	YGL191W	COX13	RCC-IV	0	No	III	B	A'	C'	D'
	YHR051W	COX6	RCC-IV	0	No	III	B	A'	C'	D'
	YIL111W	COX5B	RCC-IV	0	No	III	/	/	/	/
	YLR038C	COX12	RCC-IV	0	No	III	B	B'	B'	C'
	YLR395C	COX8	RCC-IV	0	Yes	III	B	B'	B'	C'
	YMR256C	COX7	RCC-IV	0	No	III	B	B'	B'	C'
	YNL052W	COX5A	RCC-IV	0	No	III	A	A'	A'	A'
CORE PROTEINS (MITO-ENCODED)	Q0045	COX1	RCC-IV	100,0	No	IV	No data in Tu *et al.*	/	/	/
	Q0250	COX2	RCC-IV	100,0	No	IV	No data in Tu et al.	/	/	/
	Q0275	COX3	RCC-IV	100,0	No	IV	No data in Tu et al.	/	/	/

Table S1.2: Repartition of genes involved in the COX assembly process into 6 clusters obtained using distinct clustering algorithms. *This table is also presented in the main text (**Table 1**). Here, the last three column (colored in red) show the name of the cluster in which each gene was classified using three different algorithms (hierarchical clustering "Hclust", k-means and PAM).*

References

Moloshok, T. D., R. R. Klevecz, et al. (2002). "Application of Bayesian decomposition for analysing microarray data." Bioinformatics **18**(4): 566-75.

Palumbo, M. C., L. Farina, et al. (2008). "Collective behavior in gene regulation: post-transcriptional regulation and the temporal compartmentalization of cellular cycles." Febs J **275**(10): 2364-71.

Saint-Georges, Y., M. Garcia, et al. (2008). "Yeast mitochondrial biogenesis: a role for the PUF RNA-binding protein Puf3p in mRNA localization." PLoS ONE **3**(6): e2293.

Tu, B. P., A. Kudlicki, et al. (2005). "Logic of the yeast metabolic cycle: temporal compartmentalization of cellular processes." Science **310**(5751): 1152-8.
