# Supplementary material for: Spatio-Temporal Dynamics of Yeast Mitochondrial Biogenesis: Transcriptional and Post-Transcriptional mRNA Oscillatory Modules
Source: PLoS Comput Biol. 2009 Jun 12;5(6):e1000409. doi: 10.1371/journal.pcbi.1000409 (PMC2690403; doi:10.1371/journal.pcbi.1000409)
Supplement: Text S2 — Distribution of all Puf3p targets (as determined experimentally by Gerber et al.) across the A to F phases. (0.43 MB PDF) [file pcbi.1000409.s003.pdf]

<u>Text S2</u>: Distribution of all Puf3p targets (as determined experimentally by Gerber et al.) across the A to F phases

Complete list of Puf3p targets, experimentally characterized by Gerber *et al.* (Gerber, Herschlag et al. 2004) were downloaded from the PLoS Biology web site (Table S5 - http://biology.plosjournals.org/perlserv/?request=get-document&doi=10.1371/journal.pbio.0020079).

From the 220 Puf3p mRNA targets identified by Gerber *et al.*, 170 corresponded to genes included in our set of 626 mitochondrial genes. The percentage of these 170 mRNAs in each EDPM phase is presented **Figure S2.1** (below). As expected, <u>most of the Puf3p targets were found in phase A</u> (more than 80%). Note that such a result fully agrees with our observation that the motif P3E (Foat, Houshmandi et al. 2005) is significantly overrepresented in the 3' UTR of mRNA highly expressed during phase A (the enrichment p-value = 6×10^{-120}) (**Figure 5B**).

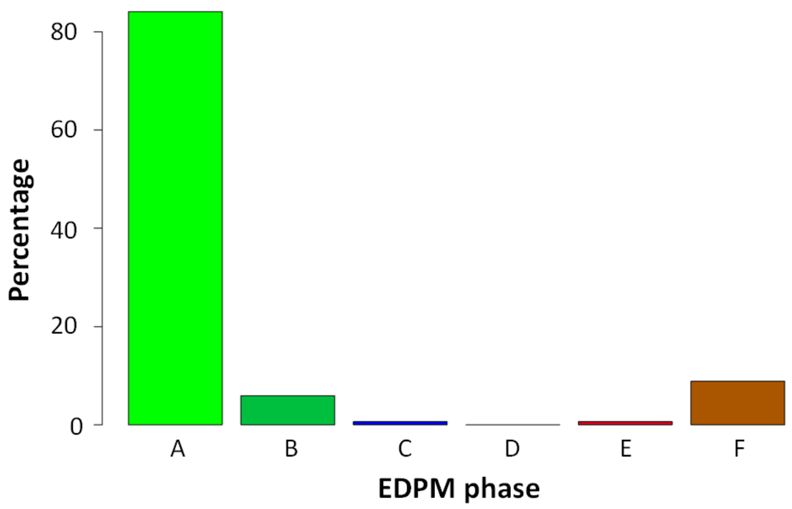

Figure S2.1: Distribution of Puf3p target genes across the A to F phases. *From the 220 genes experimentally characterized by Gerber et al. (Gerber, Herschlag et al. 2004) as being Puf3p target genes, 170 were analyzed in this study because (i) they were identified as involved in mitochondrial biogenesis, as defined by (Saint-Georges, Garcia et al. 2008) and (ii) they displayed significant periodic patterns during YMC, as defined by (Tu, Kudlicki et al. 2005). The percentage of these 170 genes, found in each EDPM temporal phases A to F is presented here. As expected, most of the Puf3p mRNA targets are found in phase A.*

Reference

Gerber, A. P., D. Herschlag, et al. (2004). "Extensive association of functionally and cytotopically related mRNAs with Puf family RNA-binding proteins in yeast." <u>PLoS Biol</u> **2**(3): E79.
